# The Common FTO Genetic Polymorphism rs9939609 is Associated with Increased BMI in Type 1 Diabetes but not with Diabetic Nephropathy

**DOI:** 10.4137/bmi.s4599

**Published:** 2010-04-27

**Authors:** Harvest F. Gu, Alexandra Alvarsson, Kerstin Brismar

**Affiliations:** Department of Molecular Medicine and Surgery, Karolinska Institutet, Karolinska University Hospital, Solna, Stockholm, Sweden. Email: harvest.gu@ki.se

**Keywords:** diabetic nephropathy, fat mass and obesity associated, genetic association, single nucleotide polymorphism

## Abstract

The fat mass and obesity associated (FTO) gene has an important genetic effect on body mass index (BMI) and risk of obesity, and obesity contributes to the progression of renal diseases, including diabetic nephropathy. We thus conducted a genetic association study to evaluate whether the FTO gene confers the risk susceptibility to the development of diabetic nephropathy. Genotyping experiments of the common FTO polymorphism, rs9939609, in 1170 type 1 diabetes patients with (n = 597) or without diabetic nephropathy (n = 573) were performed with TaqMan allelic discrimination. All subjects are of European descent and selected from the Genetics of Kidney Diseases in Diabetes (GoKinD) study. The frequency of T allele of this polymorphism was 0.414 in the studied population. There was no allelic association of this polymorphism with diabetic nephropathy. But, the risk susceptibility of A allele conferring to the increased BMI among type 1 diabetes patients was observed. The subjects carrying with AA genotype had higher BMI compared to the carriers with TA and/or TT genotype(s) (*P* ≤ 0.019). The present study provides evidence that the common FTO genetic polymorphism, rs9939609, is associated with increased BMI in type 1 diabetes but not with diabetic nephropathy.

## Introduction

Obesity, associated with insulin resistance, is a core component of the metabolic syndrome, and is among the top ten global health risks classified by the World Health Organization (WHO). Current evidence has demonstrated that obesity is associated with the early onset of glomerulomegaly, hemodynamic changes of a hyperfiltering kidney and increased albuminuria.^1^–^3^ In patients with chronic kidney disease (CKD), variation of proteinuria is associated with body mass index (BMI).^4^ Thus, obesity contributes to the progression of renal diseases, including diabetic nephropathy.

In the recent two years, several genome-wide association (GWA) studies have indicated that the fat mass and obesity associated (FTO) gene has an important genetic effect on BMI and risk of obesity. A common genetic polymorphism, rs9939609, in the FTO gene is found to associate with BMI and predisposes to childhood and adult obesity. Rs9939609 is an intronic single nucleotide polymorphism (SNP, the code is W = A/T) and resides in the first intron of the gene. The AA homozygous genotype results in an average increase of ∼3 kg in body weight or one BMI unit in the subjects compared to the TT genotype.^5^–^8^ Obviously, this FTO variant is involved in the pathogenesis of obesity. However, it is unclear whether the FTO gene confers the risk susceptibility to the development of diabetic nephropathy. Therefore, it is necessary to carry out a genetic study of the FTO gene in diabetic nephropathy. In the present study, we have conducted a genetic association study of this FTO genetic polymorphism with diabetic nephropathy in type 1 diabetes using a well-characterized material selected from Genetics of Kidney Diseases in Diabetes (GoKinD) study.^9^

## Materials and Methods

### Subjects

A total of 1170 type 1 diabetes patients with (n = 597) or without diabetic nephropathy (n = 573) were included in the present study. All subjects are of European descent and selected from the GoKinD study.^9^ The patients with diabetic nephropathy had persistent proteinuria or end-stage renal disease (ESRD) for at least 10 years. Persistent proteinuria was defined as 2 out of 3 tests of Urine Albumin/Urine Creatinine Ratio (ACR) value exceeding 300 μg albumin/mg of urine creatinine. The study was approved with the local ethics committees. Data and material transfer agreements were completed prior to the study. Information of clinical characteristics of the subjects is available as described in the previous reports.^9^,^10^

### Genotyping experiments

We have genotyped the common FTO polymorphism, rs9939609, with a standard protocol of TaqMan allelic discrimination. The assay and instrument used for allelic discrimination in genotyping experiments was purchased from Applied Biosystems (ABI 7300, Foster City, USA). For quality control, the subjects were distributed randomly across the plates with equal numbers of cases and controls on each PCR plate. Negative controls (Universal-mixture blanks) were included on each plate. Genotyping experiments in approximately 20% of samples were performed in duplicate. Successful genotype calls were ∼98% and duplication accuracy 100%.

### Statistical analyses

Proportions of genotypes or frequencies of alleles between the cases and controls were compared using a Chi-square (χ^2^) test. *P*-values <0.05 were considered as significant. Armitage’s trend, additive and dominant model tests were performed. Odds ratios (OR) and 95% confidence intervals (CI) were calculated to test the relative risk for association. The sample sizes in the GoKinD populations had ∼90% power (at the 5% level) assuming MAFs of 0.15. Statistical powers were calculated using software of PowerSampleSize (PS version 2.1.31). Clinical data are expressed as mean ± SD. Analysis of variance (one-way or two-way ANOVA) or covariance was performed to investigate differences of clinical parameters according to genotyes or groups. Non-normally distributed traits (plasma cystatin C, serum creatinine and BMI) were transformed into natural logarithms before analysis. All analyses were performed using STATISTICA version 7.1 (Statsoft, Tulsa, USA).

## Results and Discussion

We have performed a genetic association analysis of the common FTO genetic polymorphism rs9939609 in the GoKinD population and the genotype distribution is presented in Table 1. Data indicated that genotype distribution for this FTO genetic polymorphism was kept in Hardy-Weinberg equilibrium (*P* = ∼0.4). The frequency of minor allele (MAF) A was 0.414 in the studied population, and no significant difference of the MAFs between type 1 diabetes patients with and without diabetic nephropathy was found (0.411 vs. 0.416, *P*-values were 0.796, test with additive model, and 0.415, test for allele positivity, respectively).

We further analyzed clinical parameters according to the TT, TA and AA genotypes of this FTO polymorphism. Results are summarized in Table 2 and indicated that the overall type 1 diabetes subjects carrying with TT, AT and AA genotypes have significantly and gradually increased BMI (*P =* 0.019, ANOVA test; and 0.008, the comparison between TT and AA). Further analyses of cystatin and creatinine levels among the subjects carrying with TT, AT and AA genotypes with or without adjustment for BMI and/or age were performed. But, no significant difference was seen.

We have carried out a genetic association study of the common FTO genetic polymorphism, rs9939609, in diabetic nephropathy. Although there was no significant association between this polymorphism and diabetic nephropathy, type 1 diabetes patients carrying with AA genotype had significantly increased BMI compared to the patients with TT and/or AT genotype(s). Evidence has indicated that increased BMI may increase the risk for the development of diabetic nephropathy in type 1 diabetes.^11^ Recent genetic association studies have demonstrated that the FTO genetic polymorphism has no genetic effect on type 1 diabetes when the comparison analysis between type 1 diabetes patients and nondiabetic controls is performed.^12^ Furthermore, this FTO polymorphism has been identified to confer an increased risk for both type 2 diabetes and obesity. After adjustment for BMI, however, the association with type 2 diabetes is vanished because the impact of FTO on type 2 diabetes is mainly due to the association of FTO with BMI.^5^ Therefore, the present study provides evidence that the common FTO genetic polymorphism, rs9939609, is associated with increased BMI in type 1 diabetes but not with diabetic nephropathy. Taking together with other reports^5^–^8^,^11^,^12^ and the present study, we conclude that the common FTO genetic polymorphism, rs9939609, confers the risk susceptibility to the increasing BMI, but not fundamentally contributes to the development of diabetic nephropathy.

## Figures and Tables

**Table 1.** Genotype distribution of rs9939609 in USA GoKinD population.

**Group**	**N**	**TT**	**TA**	**AA**	**MAF(%)**	***P*-value**
Overall	1170	401	570	199	41.4	
T1D with DN	597	198	301	98	41.6	
T1D without DN	573	203	269	101	41.1	0.796^a^/0.415^b^

a Tests for genetic association were performed with an additive model.

b or for allele positivity.

**Abbreviations:** T1D, Type 1 diabetes; DN, Diabetic Nephropathy; MAF, Minor Allele Frequency.

**Table 2.** Clinical parameters according to the genotypes of rs9939609.

**Parameter**	**Group**	**TT**	**TA**	**AA**	***P*-value**
BMI (kg/m^2^)	Overall	25.34 ± 4.34	26.03 ± 4.99	26.43 ± 5.20	0.019^c^/0.008^d^
	T1D without DN	25.42 ± 3.65	26.35 ± 4.75	26.36 ± 4.38	0.055^c^/0.056^d^
	T1D with DN	25.25 ± 4.93	25.67 ± 5.23	26.50 ± 5.91	0.060^c^/0.057^d^
Cystatin (mg/L)	Overall	1.51 ± 1.34	1.56 ± 1.40	1.58 ± 1.47	0.826^c^/0.567^d^
	T1D without DN	0.81 ± 0.13	0.81 ± 0.14	0.81 ± 0.13	0.932^c^/0.832^d^
	T1D with DN	2.20 ± 1.60	2.36 ± 1.67	2.35 ± 1.77	0.580^c^/0.454^d^
Creatinine (mg/dL)	Overall	1.50 ± 1.43	1.50 ± 1.45	1.51 ± 1.39	0.997^c^/0.913^d^
	T1D without DN	0.90 ± 0.16	0.89 ± 0.16	0.88 ± 0.16	0.603^c^/0.608^d^
	T1D with DN	2.10 ± 1.84	2.18 ± 1.88	2.13 ± 1.76	0.908^c^/0.882^d^

c Tests for phenotype difference were done with ANOVA.

d or by the comparison between TT and AA.

**Abbreviations:** T1D, Type 1 diabetes; DN, Diabetic Nephropathy. Data of clinical parameters are mean ± SD.

## Abbreviations

ACR: Albumin/urine creatinine ratio
ESRD: End-stage renal disease
DN: Diabetic nephropathy
FTO: Fat mass and obesity associated
GoKinD: Genetics of Kidney Diseases in Diabetes study
MAFs: Minor allele frequencies
SNP: Single nucleotide polymorphism
T1D: Type 1 diabetes
T2D: Type 2 diabetes
